# Contribution of Apolipoprotein L1 (*APOL1*) Risk Alleles to Kidney Disease in West Africa: an opportunity for treatment

**DOI:** 10.4314/gmj.v58i4.1

**Published:** 2024-12

**Authors:** Dwomoa Adu

**Affiliations:** University of Ghana Medical School Accra, Ghana

**Keywords:** Chronic kidney disease, Apolipoprotein L1, Treatment

Chronic kidney disease (CKD) is common in sub-Saharan Africa (SSA) and develops in 10-14 per cent of adults.^1^,^2^ CKD leads, in many cases, to ill health, kidney failure, and premature death. The increased prevalence of CKD in Africa is in part due to inherited variants in the gene for Apolipoprotein L1 (APOL1). These variants developed some 10,000 years ago, and because they provided protection against fatal sleeping sickness (*Trypanosoma brucei rhodesiense*), the proportion of individuals with these variants rose to a high level in Africans in SSA.^3^ The *APOL1* variants are associated with the development of CKD in Africans and people of African descent. The three fold increased risk of end stage kidney disease (ESKD) in African Americans compared to European Americans is now attributed largely to variants in the *APOL1* gene in chromosome 22q12 locus, termed G1 and G2.^4^,^5^ The impact of the gene variants mirrors other evolutionary adaptations found in African populations. In this way, the gene is like the sickle cell gene, which protects people against malaria but can cause crises.

Our recently published research in the New England Journal of Medicine identified that the APOL1 variants were a major genetic factor contributing to kidney disease among West African populations.^6^ This case-control study was conducted by the Human Health and Heredity in Africa (H3Africa) Kidney Disease Research Network a Consortium.^7^ We recruited 8,355 individuals (4712 subjects with CKD (Stages 2-5), 866 with biopsy-proven glomerulonephritis and 2777 subjects with no kidney disease from Ghana and Nigeria) across 13 sites (two in Ghana and 11 in Nigeria). Blood and urine samples were collected from these subjects. In the study population, 29.7% had two *APOL1* risk alleles, and 43% had one *APOL1* risk allele. Compared to subjects with one or no risk alleles, two copies of the *APOL1* risk allele increased the odds ratio of kidney disease by a quarter (25%), odds ratio, 1.25; 95% confidence interval [CI], 1.11 to 1.40. Participants with one risk allele had an increased risk of CKD of 18% compared to subjects with no risk allele with an adjusted odds ratio of 1.18: 95% CI, 1.04 to 1.33. What once served as a protective mechanism against a deadly disease, Trypanosomiasis, has become a significant contributing factor to kidney disease in contemporary times. The implications of this research extend far beyond West Africa and include populations of African descent worldwide.

Recently, a company called Vertex have developed a drug, Inaxaplin, that blocks the pore-forming action of *APOL1* variants. A study^8^ showed that patients treated with two *APOL1* treated with Inaxaplin reduced their proteinuria after 13 weeks. We have embarked on a trial of Inaxaplin in patients aged 12 to 65 years with CKD, not due to diabetes or lupus nephritis. The inclusion criteria are an estimated glomerular filtration rate (eGFR) ≥25 to < 75 mL/min/1.73 m^2^ and a urine protein creatinine ratio ≥0.7 g/g to <10g/g.
